# Long-term effects of weak electrical stimulation on active neuronal networks

**DOI:** 10.1186/1471-2202-14-S1-P308

**Published:** 2013-07-08

**Authors:** Davide Reato, Marom Bikson, Lucas C Parra

**Affiliations:** 1 Department of Biomedical Engineering, The City College of the City University of New York, New York, NY, 10031, USA

## 

Transcranial direct-current stimulation induces cortically sub-millivolt electric fields that modulate brain activity and induce long-term (plastic) effects measurable as improved cognitive and behavioral performances in human experiments [1]. However, the basic mechanisms by which a weak electric field can induce long term effects are not clear yet. This is a limitation for developing more targeted stimulation protocols or to maximize the outcome of the stimulation. In particular, considering that 1 V/m electric field can polarize the neuronal membrane at most 0.2 mV [2], it is still a mystery how such a small voltage fluctuation can mediate any significant long-term effect. Here we combine experiments in rat brain slices and computational models of neuronal networks to determine how fields can induce long-term effects. Our hypothesis is that basal neuronal depolarization and network activity are required to amplify the effects of the electric field and so to mediate plasticity. Network activity can be induced in hippocampal rat slices by applying carbachol, a cholinergic agonist [3]. The coherent network activity can be measured extracellularly in the high beta/low gamma frequency band. The activity persists for many hours and is generated from the interplay of excitation of pyramidal neurons and inhibitory feedback. We have previously characterized the effects of fields on gamma oscillations during stimulation [4]. Gamma power is modulated depending on the frequency and amplitude of the stimulation. A computational model based on Izhikevich's single neuron dynamics describing synaptically connected excitatory and inhibitory neurons was parameterized based on the results of the extracellular recordings in slices. The model explained the effects of the electric field on firing rate and spike timing and made precise predictions about intracellular experiments that were confirmed. However, how these acute effects of stimulation translate to long-term was not investigated. Previous experimental and theoretical studies have shown that small injected currents on post-synaptic neurons can strongly modulate LTP/LTD induction in paired pre- and post-synaptic neurons intracellular recordings and even result in plastic effects despite timing discrepancy between the two spike timings [5,6]. The effects are thought to be mediated by dendritic depolarization [6]. Based on these results and considering that weak electrical stimulation induce small membrane polarizations, we investigate how small currents can induce long term plastic effect. We applied DC electric fields for 10 minutes during carbachol-induced gamma oscillations in hippocampal brain slices and record the oscillations for 1 hour after stimulation. The basal depolarization induced by carbachol (~10 mV) is a range that is plasticity-permissive. To further investigate how electric fields affect neuronal somatic and dendritic compartments, we use current source density to estimate sink and source dynamics during oscillations and stimulation. Our preliminary results show that indeed, electrical stimulation not only acutely modulates the power of the oscillations but also produces after-stimulation effects. We also perfuse slices with synaptic blockers to determine which receptors mediate possible long-term effects induced by the electrical stimulation. The experimental results are then combined to model the effect of electric field on gamma oscillations and plastic synapses at a network level.
